# A novel mutation in the *COL2A1* gene in a patient with Stickler syndrome type 1: a case report and review of the literature

**DOI:** 10.1186/s13256-017-1396-y

**Published:** 2017-08-26

**Authors:** Yousuke Higuchi, Kosei Hasegawa, Miho Yamashita, Hiroyuki Tanaka, Hirokazu Tsukahara

**Affiliations:** 1 Department of Pediatrics, Okayama University Graduate School of Medicine, Dentistry and Pharmaceutical Sciences, 2-5-1 Shikata-cho, Kita-ku, Okayama, 700-8558 Japan; 2 Department of Pediatrics, Okayama University Hospital, 2-5-1 Shikata-cho, Kita-ku, Okayama, 700-8558 Japan; 3 Faculty of Human Life Sciences, Notre Dame Seishin University, 9-16-2 Ifuku-cho, Okayama, 700-8516 Japan; 4 Department of Pediatrics, Okayama Saiseikai General Hospital, 1-7-18 Ifuku-cho, Kita-ku, Okayama, 700-8511 Japan

**Keywords:** Stickler syndrome, *COL2A1*, Type II collagenopathy, Marshall syndrome

## Abstract

**Background:**

Stickler syndrome is a group of collagenopathies characterized by ophthalmic, skeletal, and orofacial abnormalities, with the degree of symptoms varying among patients. Mutations in the *COL2A1*, *COL11A1*, and *COL11A2* procollagen genes cause Stickler syndrome. Marshall syndrome, caused by a *COL11A1* mutation, has clinical overlap with Stickler syndrome.

**Case presentation:**

A 2-year-old Japanese boy was presented to our hospital with short stature (79.1 cm, −2.52 standard deviation). His past medical history was significant for soft cleft palate and bilateral cataracts. He had a flat midface, micrognathia, and limitations in bilateral elbow flexion. Radiographs showed mild spondyloepiphyseal dysplasia. Initially, we suspected Marshall syndrome, but no mutation was identified in *COL11A1*. At 8 years old, his height was 116.2 cm (−1.89 standard deviation), and his orofacial characteristics appeared unremarkable. We analyzed the *COL2A1* gene and found a novel heterozygous mutation (c.1142 G > A, p.Gly381Asp).

**Conclusions:**

In this case report, we identify a novel missense mutation in the *COL2A1* gene in a patient with Stickler syndrome type 1, and we describe age-related changes in the clinical phenotype with regard to orofacial characteristics and height. Genetic analysis is helpful for the diagnosis of this clinically variable and genetically heterogeneous disorder.

## Background

Stickler syndrome is a group of hereditary conditions characterized by high myopia, retinal detachment, cataracts, sensorineural or conductive hearing loss, mild spondyloepiphyseal dysplasia, early-onset osteoarthritis, midfacial underdevelopment, and cleft palate (as part of the Pierre Robin sequence). The degree of these symptoms varies among individuals. The incidence of Stickler syndrome is estimated to be approximately 1 in 7500–9000 neonates [1]. Stickler syndrome is a genetically heterogeneous disorder caused by abnormal synthesis of type II, XI, or IX collagen. Stickler syndrome type 1 (STL1) [MIM:108300] is the most common type, caused by mutations in the *COL2A1* [MIM:120140] gene on chromosome 12q13.11. Stickler syndrome type 2 [MIM:604841] is the second most common, caused by mutations in the *COL11A1* gene [MIM:120280] on chromosome 1p21. Stickler syndrome type 3 (﻿a﻿utosomal dominant otospondylomegaepiphyseal dysplasia)[MIM:184840] is caused by mutations in the *COL11A2* gene [MIM:120290] on chromosome 6p21.3. Stickler syndrome type 3 presents with characteristics similar to those of STL1 and Stickler syndrome type 2, except for the lack of ocular manifestations. These three types of Stickler syndrome show autosomal dominant heredity. Autosomal recessive Stickler syndrome has been reported in rare cases with mutations in collagen IX genes [2]. *COL2A1* encodes an alpha-1 chain procollagen monomer that assembles into a homotrimer. These stable triple helices aggregate into fibrillar collagen (type II collagen). Type XI collagen is also fibrillar collagen and regulates the fibril diameter of type II collagen. Type IX collagen is a fibril-associated collagen with interrupted triple helices. Types II, XI, and IX collagen are expressed in the same tissues, which include cartilage, vitreous humor, intervertebral discs, and the inner ear.

Marshall syndrome [MIM:154780], which is caused by mutations in *COL11A1*, has a clinical overlap with Stickler syndrome [3]. Distinguishing between Stickler syndrome and Marshall syndrome is not easy. It has been suggested that people with Marshall syndrome more often have a short stature, early-onset hearing loss, and more pronounced maxillary hypoplasia [4–6]. In this report, we describe a case of a patient with STL1 with a novel mutation in the *COL2A1* gene.

## Case presentation

A 24-month-old Japanese boy was referred to our hospital for evaluation of his short stature. He was born at a gestational age of 38 weeks and 4 days and had a submucous cleft palate and micrognathia. His birth weight was 3225 g (+0.53 standard deviation (SD) of average Japanese male infants), and his birth length was 45 cm (−1.80 SD). His submucous cleft palate was repaired at 18 months of age. Tympanostomy tubes were placed at the same time for secretory otitis media. Auditory brainstem response testing showed normal findings. At 22 months, he was found to have bilateral leukocoria. Phacoemulsification and anterior vitrectomy for bilateral lens opacities were performed at another hospital. He was considered to have Pierre Robin sequence, but the association with the ophthalmic symptoms was not investigated. He received speech therapy for speech delay.

**Fig. 1:**
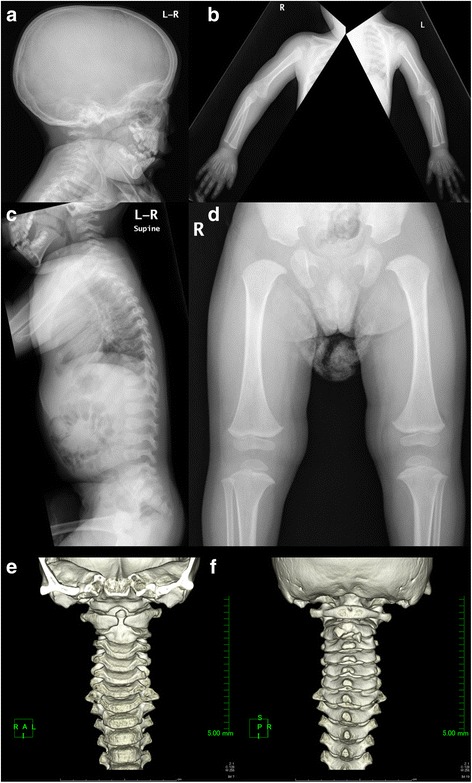
Radiological images of the patient. Radiographic images show thickening of the calvaria (**a**); metaphyseal flaring of the distal humerus (**b**); mild kyphosis (**c**); and lack of femoral head ossification, deformity of the femoral neck, and distal femoral and proximal tibial epiphyseal ossification centers (**d**). Reformatted computed tomographic scans of the cervical spine show hypoplasia of dens and separation of the anterior atlas arch (**e**), as well as abnormal ossification at the spinous process of the axis (**f**)

At presentation, the patient weighed 11.6 kg (−0.33 SD) with a height of 79.1 cm (−2.52 SD). His arm span was 79 cm, and his sitting height was 48.5 cm (sitting height/height ratio 0.61). He showed ocular hypertelorism; prominent eyes due to shallow eye sockets; a flat midface with a depressed nasal bridge; anteverted nares; micrognathia; low-set ears; and limitations in bilateral elbow flexion; but no hypotonia, hyperextensible joints, or joint pain. All other family members, which included his parents and two brothers, were healthy. His parents are not consanguineous. His father’s height was 182 cm (+1.93 SD of average Japanese adult men), and his mother’s height was 147 cm (−2.09 SD of average Japanese adult women). Radiographs showed thickening of the calvaria, widening of the distal humeral metaphysis, lack of femoral head ossification, deformity of the femoral neck, and distal femoral and proximal tibial epiphyseal ossification centers. These features suggested mild spondyloepiphyseal dysplasia (Fig. 1a–d). Serum insulin-like growth factor-1 was 20.17 nmol/L (154 ng/ml) (normal range 2.36–20.17 nmol/L [18–154 ng/ml]). An L-arginine stimulation test for growth hormone (0.5 g/kg arginine) showed a peak growth hormone level of 16.4 ng/ml. A luteinizing hormone-releasing hormone/thyrotropin-releasing hormone stimulation test (3 μg/kg luteinizing hormone-releasing hormone; 10 μg/kg thyrotropin-releasing hormone) showed prepubertal and normal thyroid-stimulating hormone responses. His karyotype was 46,XY. A brain magnetic resonance imaging scan showed no abnormalities. Echocardiography showed no evidence of mitral valve prolapse. At that time, we suspected Marshall syndrome, but no mutation was identified in exons or exon/intron boundaries of the *COL11A1* gene.

**Fig. 2:**
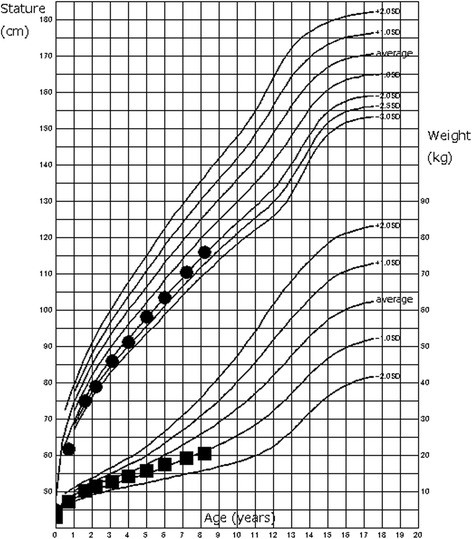
Growth chart. The patient’s short stature gradually improved over time

**Fig. 3:**
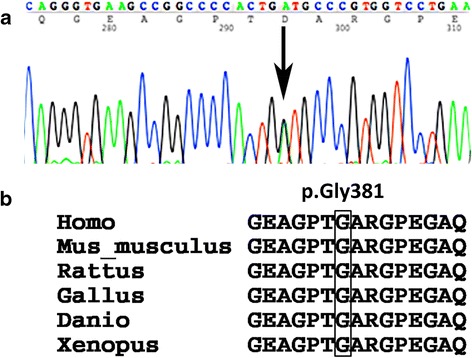
Mutation analysis of the *COL2A1* gene. Heterozygous mutation c.1142G > A (p.Gly381Asp) is denoted by the *arrow*. The glycine substitution mutation is in the Gly-X-Y triplet repeats region of the type II collagen triple helix (**a**). The glycine at amino acid 381 is highly conserved throughout species (**b**)

At the age of 8 years, the patient’s height was 116.2 cm (−1.89 SD) (Fig. 2), and his orofacial characteristics appeared unremarkable. Although 3D reformatted computed tomographic scans showed atlantoaxial dysplasia, he had no clinical symptoms of atlantoaxial instability (Fig. 1e and f). He developed no symptoms of retinal detachment, deafness, or osteoarthritis. The patient studied at a normal elementary school and his mental development was normal. We next suspected Stickler syndrome and conducted *COL2A1* genetic analysis. Genomic deoxyribonucleic acid was extracted from the patient’s peripheral blood. Polymerase chain reaction primers were designed to amplify all coding exons and exon/intron boundaries of the *COL2A1* gene. Primer sequences are available upon request. Sequence analysis showed a novel heterozygous mutation in exon 19 of *COL2A1*, c.1142G > A (p.Gly381Asp) (Fig. 3a), and no other mutation was identified. The glycine at amino acid 381 was located in the Gly-X-Y triplet repeats region of the type II collagen triple helix, and it is phylogenetically conserved throughout species (Fig. 3b) [7]. This variant has not been described in the Single Nucleotide Polymorphism database, the 1000 Genomes Project, Leiden Open Variation Database 3.0, or the Human Genetic Variation Database in Japanese. *In silico* analysis showed that this mutation was predicted to be “probably damaging” by PolyPhen-2 (score 0.999) and to be “disease causing” by MutationTaster (score 94) [8, 9].

## Discussion

In this case report, we identify a novel substitution of glycine within the Gly-X-Y triplet repeats region of the type II collagen triple helix. Authors of several studies have reported that most mutations that cause type II collagenopathy are distributed in the Gly-X-Y triplet repeats region, and there is no hot spot for mutations in the *COL2A1* gene [1, 10, 11]. Glycine replacement within the Gly-X-Y repeat accounts for many of the mutations and confirms that this type of gene alteration predominates in type II collagenopathies [10, 11]. The changes elicit structural alterations of half of the alpha-1 chains and result in abnormal conformation and destabilization of the triple helix [11, 12]. We therefore concluded that this mutation was responsible for this patient’s phenotype.

Heterozygous mutations in the *COL2A1* gene cause a spectrum of type II collagenopathies, including those that are lethal in neonates, such as achondrogenesis type II/hypochondrogenesis [MIM:200610], and nonlethal conditions, such as spondyloepiphyseal dysplasia congenital [MIM:183900]; spondyloperipheral dysplasia [MIM:271700]; spondyloepimetaphyseal dysplasia, Strudwick type [MIM:184250]; Kniest dysplasia [MIM:156550]; and Stickler syndrome. Ophthalmic, auditory, skeletal, and orofacial abnormalities are shared with these nonlethal type II collagenopathies; however, short trunk dwarfism is common in all but Stickler syndrome [1, 10]. Our patient’s short stature was mild and not disproportionate [13, 14]. Furthermore, orofacial features were not evident, and his short stature improved with age. Characteristics of midfacial underdevelopment in Stickler syndrome are more pronounced in infants and young children, and they become less distinctive with age [15, 16]. In the majority of patients with Stickler syndrome, height is within the normal range, but the growth pattern is uncertain [17]. The phenotype of Stickler syndrome seems to become less distinct with increasing age, similar to quantitative or qualitative defects in type I collagen (for example, osteogenesis imperfecta) [18]. Premature stop (nonsense, frameshift, or splicing) mutations of *COL2A1* usually result in STL1, whereas glycine substitutions in the triple-helix region are typically associated with lethal (such as achondrogenesis type 2/hypochondrogenesis) or more severe short stature (such as spondyloepiphyseal dysplasia congenital, Kniest dysplasia). The location of the glycine substitution or the nature of the substituting amino acid seems to affect the skeletal phenotype; however, there is no clear genotype–phenotype correlation [1, 10, 11]. On the basis of our clinical findings, the patient was diagnosed with STL1 [11].

Patients with Stickler syndrome exhibit varying signs and symptoms, which often may result in a delayed or missed diagnosis [17]. Some clinical diagnostic criteria for STL1 have been proposed, but they have not been validated [1, 19]. Authors of several studies have reported that Stickler syndrome is the basis for about 20–50% of infants with Pierre Robin sequence [20, 21]. It was suggested that Stickler syndrome should be considered in any neonates with Pierre Robin sequence, particularly in those with a family history of cleft palate and in patients with dominantly inherited myopia, nontraumatic retinal detachment, and/or mild spondyloepiphyseal dysplasia [22]. Accurate diagnosis is important for the assessment and management of ophthalmic, auditory, and articular manifestations, which are sometimes not obvious, and also for genetic counseling.

Marshall syndrome is similar to Stickler syndrome, and there has been debate over whether Stickler syndrome and Marshall syndrome are distinct conditions [4, 17, 23]. Genetic analysis has revealed that both diseases are allelic disorders caused by mutations in the *COL11A1* gene [5, 6]. Most patients with Marshall syndrome have a splice-site mutation in the *COL11A1* gene [3]. A short nose, anteverted nares, midfacial hypoplasia, and a flat nasal bridge are common in patients with Marshall syndrome and are also seen in young patients with mutations in the *COL2A1* gene (STL1) [5]. We first considered a diagnosis of Marshall syndrome, but molecular analysis confirmed a diagnosis of STL1.

## Conclusions

We identified a novel missense mutation in the *COL2A1* gene in a patient with STL1, and we report age-related changes in the clinical phenotype with regard to orofacial characteristics and height. Genetic analysis is helpful for the diagnosis of this clinically variable and genetically heterogeneous disorder.

## Abbreviation

STL1: Stickler syndrome type 1
